# *“You have to walk on eggshells around him”*: Female partners’ perspectives on the opportunities and challenges of dealing with a male partner’s depression—A qualitative study

**DOI:** 10.1371/journal.pone.0336209

**Published:** 2025-11-07

**Authors:** Maja Stiawa, Paul Nickel, Gironimo Krieg, Katharina Senk, Reinhold Kilian, Natalie Lamp, Maria Panzirsch, Silvia Krumm

**Affiliations:** 1 Department for Psychiatry and Psychotherapy II, Ulm University, Ulm, Germany; 2 Department of Psychiatry I, Ulm University, ZfP Südwürttemberg, Germany,; 3 Department of Psychiatry and Psychotherapy, University of Leipzig Medical Center, Leipzig, Germany,; 4 Department for Psychiatry, Psychotherapy and Psychosomatics, District Hospital Donauwörth, Donauwörth, Germany; King George's Medical University, INDIA

## Abstract

**Purpose:**

Partners of people with mental illness can play an important role in helping them cope with the illness. Previous studies have highlighted the potential burden of depression on partners, but there has been little research into the perspective of female partners of men with depression in terms of their role and support needs. The aim of this study is to investigate the subjective view of female partners on the opportunities and challenges of dealing with a male partner’s depression.

**Materials and methods:**

Qualitative semi-structured interviews were conducted online with 13 female partners of men with depression using a semi-structured interview guide. The transcripts were analyzed using qualitative content analysis.

**Results:**

Four central categories were derived from the analysis: 1) perceived changes in depressed partners; 2) positive impacts on their relationship; 3) burdens and challenges in coping with the partner’s depression; and 4) experiences with and expectations of support. Female partners often take on an active and demanding role during the depression treatment period and exhibit a high level of caring behavior towards and responsibility for their partner with depression. Compensating for the impact of the partner’s depression on family life, women have to bear additional hardship that may lead to overload. The women’s needs are primarily met by close contacts from within their personal networks. Joint therapy sessions during the male partner’s treatment are valuable in helping cope with the illness together.

**Conclusions:**

For partners of men with depression, the impact of the illness is ambivalent: On the one hand, the partner’s depression is associated with a number of difficulties and challenges. On the other hand, dealing with the illness together can also strengthen their relationship. Female partners of men treated for depression should be provided with low-threshold services, including therapeutic interventions that focus on successful joint coping.

## Introduction

Partners of people with mental illness can be an important source of support, providing emotional support, useful information, and concrete assistance [1,2]. Regardless of the support that partners can potentially provide for the person with mental illness, studies have indicated that partners and family members of mentally ill persons may face a number of challenges related to the mental illness, have a lower quality of life, and poorer mental health [3–5]. Studies on the condition of partners have shown significantly lower levels of psychological well-being [3,4] and number of social relationships [4] as well as higher instances of anxiety and depression [3,5] when compared to the general population. Findings from a qualitative study in Norway that explored the daily life experiences of partners, parents, and children of inpatients with depression showed the high burden on relatives due to the combination of being simultaneously responsible for the person with depression, their family, and their own job. These challenges are often complemented by fears about the future and financial worries [6]. As a result of these various burdens, partners may develop strong feelings of helplessness, frustration, and anger during the period of their partner’s illness; in such situations, a support network could assist in helping them better manage their situation [2]. Likewise, Priestley et al. [2018] described the partial social withdrawal of couples in which one partner suffers from chronic depression due to their awareness of social stigma. This may be an additional difficulty for affected couples, especially with regard to the support required by couples who have to cope with the illness over a long period of time. Behind the background of the long-term burden such couples face, the authors emphasized the need for comprehensive support in partners of people with mental illness [7].

Psychoeducation and partner therapy are services that can provide support to partners in assessing their ill partner’s behavior and the significance of their own reaction [2,7]. Skundberg-Kletthagen et al. concluded that interdisciplinary and individual support services involving relatives are necessary in order to support the recovery of the person who is unwell and to prevent relatives’ own health from being impacted as a result of the burden [6]. However, a systematic review of the experience of relatives and partners of people with depression with professional mental health care showed that for the most part, participants experienced a lack of resources, felt excluded from the treatment process, and were not provided with sufficient information around how to handle the situation of living with a loved one with depression [8].

Previous studies that examined the condition of partners of people affected by depression have predominantly focused on the male partners of women with depression [9], or included partners of both sexes [2,7] or family members [6]. However, results from studies concerning male partners or partners of both sexes may not be applicable to female partners of men with depression due to gender-specific behaviors in both the person experiencing the illness and the partner [9]: Previous gender-sensitive studies have shown that men with depression are less likely than women to seek treatment and to begin it in a timely manner [10,11]. In line with the concept of hegemonic masculinity [12], men tend to align with norms valuing strength and independence while rejecting behaviors seen as weak and dependent. A systematic review highlighted the negative effects of such norms on men’s experience of depression and help-seeking behavior [13]. Other systematic reviews found that men associate depression with weakness and a failure to fulfill masculine ideals, indicating internalized stigma [14,15].Mental health professionals in inpatient care encourage men to revise gender-role- attitudes and health behaviors [16]. However, recent studies show that men manage depression in diverse ways shaped by gender norms that only partly reflect traditional masculinity [17–19].

To date, little is known about the role of female partners in this process. Results of a study comparing couples with and without one partner experiencing severe depression discovered gender-specific differences in thinking styles and perceptions of the illness, with female spouses of men with depression reporting a poorer quality of marital relationship than male spouses with mental illness or couples in which neither partner is experiencing mental illness [20]. A qualitative study examining the experiences of female partners of depressed men showed that the partners navigate between the expectations that females feel regarding caring for the depressed partner and their need for self-care and detachment in order to cope with the hardship resulting from the illness [21]. In order to provide tailored services for female partners of men with depression, it is important to better understand their perspectives and needs concerning the illness. Therefore, the aim of this study is to explore the subjective view of female partners concerning their male partners’ depression and its consequences with regard to their relationship to their partner and their own ways of dealing with their partner’s depression. Based on this aim, the study seeks to answer the following research questions: How do female partners of men with depression perceive and describe the impact of the illness on their relationship and their own coping? And how do they assess and respond to support services available to them?

## Materials and methods

This qualitative study was part of the mixed method study “Transformation of masculinity orientations and work-related attitudes among depressed men (TRANSMODE)” [22], funded by the German Research Foundation. The study was approved by the Ulm University ethics committee (Nr. 347/21).

### Recruitment

Male participants in the TRANSMODE study who were in a relationship and had given permission to contact their partner (n = 25) were identified. Their female partners were subsequently invited to participate in the study and were informed about study procedures via e-mail and telephone. Of the female partners contacted, eleven stated that they had no time or interest, or an interview appointment, although rescheduled several times, ultimately did not take place. The recruitment period for this study started on December first, 2023 and ended on January 15th, 2024.

Female partners who agreed to participate (n = 13) received detailed written information, the consent form, and the reimbursement form via e-mail. Participants provided written informed consent before the start of the interview. Participants received compensation totaling 30 Euro.

### Data collection

Problem-centered interviews (PCI) [23] were conducted with female partners of men with depression using a semi-structured interview guide and were concerned with the following themes: a) beginning of treatment and a subjective theory of illness; b) the couples’ strategies for coping with the illness; c) the course of the illness; d) the partner’s self-care and support; e) social networks and disclosure; and f) gender and society (S1 Table). The PCI [23] is well-suited to exploring the perspectives of female partners of men with depresion, as it combines a theory-driven focus on specific issues with openness to participants’ own interpretations. This method enables a nuanced understanding of how gendered relationship norms shape the experience and negotiation of depression within intimate partnerships.

The interviews lasted on average 53 minutes (SD = 14.7, range = 29–90). Interviews were conducted online via Zoom by MS, PN, and GK. All interviewers are experienced in conducting qualitative interviews. After conducting an interview, the interviewer wrote a short interview protocol. All interviews were recorded in audio and video form and subsequently transcribed in German by the AI-based programme f4x, which complies with the German General Data Protection Regulation (“DSGVO”). Afterwards, all transcripts were checked for accuracy by a research assistant (KS).

### Analysis

Qualitative content analysis was performed in line with Kuckartz [24] using MAXQDA software. Qualitative content analysis, as a widely used method for analyzing interview data, enables a structured yet flexible identification of recurring themes [24]. This approach allowed us to explore the female partners’ subjective perspectives on male depression while preserving the contextual depth of their accounts. As a first step, case summaries were written for each individual case, structured around the main categories defined by the interview guide (MS, PN). The interview guide served as an initial template for the first categories [24], enabling deductive coding. Deductive codes were complemented by inductive codes. According to Kuckartz [24], deductive categories are derived from existing theories or prior knowledge of the researcher and can be defined before data alalysis, whereas inductive categories only emerge from the data itself during the coding process. Analysis was performed by a senior researcher (MS), a student doctor (PN), and a student research assistant (KS). The researchers are part of the study team that conducted the present study. This is a mixed-gender group of German researchers, academically socialized within the discipline of German sociology and psychiatry, with many years of experience conducting research in the field of social psychiatry with a focus on gender-specific issues. The interdisciplinary nature of the team enabled critical discussions and triangulation during data interpretation. At the beginning of the analysis, categories were developed on the basis of three interviews (21% of all interviews). Subsequently, after three interviews had been independently coded by two researchers (MS, PN), preliminary results were regularly discussed in team meetings. In the course of the analysis, the category system was further developed and small changes such as the addition of individual inductive categories were made. All transcripts were coded again using the final category system and any additions and changes made were discussed (by KS, MS). Finally, categories regarding female partners’ description of challenges and use of support services were further developed through additional subcodes. The development of the interview guide and the progression of analysis and preliminary results were presented and discussed in qualitative research workshops at the Department for Psychiatry II at Ulm University. The analysis was carried out using the original German-language transcripts.

### Sample

The participants’ average age was 40 years (SD = 12.1, range = 19–63). The average relationship duration was 18 years (SD = 13.1, range = 3–38). More than a half were married (69.2%). All but one participant lived with their partner. Six of the 13 participants had children, with five participants having children under 18 years of age. On average, participants had a high level of education and nine out of 13 were employed (Table 1).

**Table 1:** Participant characteristics (n = 13).

	M (SD)
Age (years)	40 (12.1)
Duration Relationship (years)	18 (13.1)
**Characteristic**	**n (%)**
Marital status	
* Married*	9 (69.2)
* Partnered*	4 (30.8)
Children	
* Yes*	7 (53.8)
* -Younger than 18 years*	5 (38.5)
Living situation	
* Living apart*	1 (7.7)
* Living together*	12 (92.3)
Highest level of education	
* Middle school*	5 (38.5)
* High school*	3 (23.1)
* University degree*	5 (38.5)
Employment situation	
* Employed*	9 (69.2)
* Education/study*	1 (7.7)
* Retired*	1 (7.7)
* Unemployed*	1 (7.7)
* Other*	1 (7.7)

Note. Percentages may not sum to 100 due to rounding.

## Results

The following four central categories were derived from the analysis: 1) gendered perceptions of men’s depression symptoms; 2) positive impacts on relationship, 3) burdens and challenges in coping with the partner’s depression; and 4) experiences with and expectations of support (Table 2). The following sections provide an in-depth description of these categories, presented in the order listed above and supported by illustrative quotations.

**Table 2:** Coding tree.

Code	Subcode	*Subcode*	Quantity	
			*Nr. of Persons*	*Nr. of Codings*
**Perceived Changes in depressed Partners**	Symptom development	*Changes in behavior/ development of symptoms*	4	6
		*Symptoms „Male depression“*	5	10
		*„Typical“ Symptoms*	9	19
	Becoming aware of the diagnosis	*Own conclusion from diagnosis*	9	17
		*Disclosure through partner*	10	12
**Positive impacts on relationship**	(Changed) perception of depression		12	15
	Changed patterns of communication		9	24
	Relationship more close/ intensified		6	7
**Burdens and challenges in coping with the partner’s depression**	Partner’s path to therapy	*Role of partner at treatment start*	12	30
		*Role of other persons at treatment start*	2	2
		*Initiation of treatment/ first contact*	12	17
		*Motivators for treatment*	13	19
		*Barriers to treatment*	6	7
	Seeking help instead of/ before starting treatment		2	2
	Tipping point		9	10
	Lack of understanding		11	41
	Concerns due to the clinical picture and the consequences		9	17
	Financial burden		2	6
	Monitoring the partner		5	5
	Shouldering more tasks		6	11
	Reflecting and adjusting the own behavior		7	11
	Thoughts of Separation		5	17
**Experiences with and expectations of support**	Couple’s talk during therapy		12	34
	Professional support services	*Barriers/ Reasons for not using offers*	12	18
		*Reasons why offers are helpful/taken up/conceivable*	8	25
	Social network support		11	26

### 1. Gendered perceptions of men’s depression symptoms

Most participants described becoming aware of the illness because of the symptoms they gradually noticed in their partners. When describing behaviors in male partners during the onset period of their depression, participants reported in retrospect a wide range of symptoms, such as social withdrawal, disturbed sleep, joylessness, or rumination, as well as impulsive and aggressive behavior, confrontational behavior, and tantrums.

*“And actually, I would say it happened relatively quickly. It wasn’t such a gradual process over a long period, but things got much worse quite fast — like a lack of drive, he couldn’t feel joy anymore. That was also quite hard, because normally he would enjoy things like cooking together or eating, but suddenly there was no joy in that, no more laughter. And I think that was also when, for the first time, suicidal thoughts came up — that was in 2021, in the fall. And yes, that’s how I experienced it — it was really noticeable, or I’d say it changed quickly. It was also very weather-dependent, I’d say. Especially during those phases in November, when it’s dark a lot and the weather is bad.” (03, pos. 4)*

Participants described general differences between men and women in the way they deal with the illness. In contrast to men, participants assumed that women are more likely to seek help and to be more open in their approach regarding the depression in general. One participant assumed that *“it* [the depression] *is more noticeable in women”* (03, pos. 96).

When reflecting on whether the disease influences how they see their partner “as a man,” some participants rejected the idea that the illness affects their perception of their partner. However, some participants interpret their partner’s coping behaviors using the language of strength and weakness:

*“Yes, much weaker than usual. For sure. As I said, all he does is moan.” (13, pos. 103)*

Stereotypical norms of masculinity often served as a point of reference for participants to describe their male partners. In this sense, the following quote illustrates the participant’s critical stance towards traditional masculinity norms, while emphasizing a more positive alternative form of masculinity. A key trait of this alternative masculinity is a hightened capacity for empathy, which benefits both the partner and the children, but may also contribute to an increased vulnerability to depression:

*“Again, yes, and even more sensitive than I was aware of, but I see that mainly as a strength, because for me this ambitious perseverance, of gritting one’s teeth and pushing through has no great benefit for me as a way of life. [...] And so I also have such a good friend and partner in him, because he is open to conversations and to my needs and has adapted to this change of perspective so extremely well that he knows what is important for the other person in a given moment or what is nice for the children –and so on. I think that’s a strength of someone who is more sensitive and who is therefore unfortunately also susceptible to things like depression, I think.” (07, pos. 106)*

The subsequent quote highlights the participant’s association of depression and help-seeking with weakness, in line with hegemonic masculinity norms, while simultaneously reframing treatment-seeking as a demostration of strength:

*“In general, the process that he goes through. He comes across stronger to me. If he learns to deal with it* [the depression] *and he’s committed to being able to deal with it, and he learns to open up, that just shows me that he’s stronger than he perhaps admits to himself. […] That was a big thing, that he really mustered up the courage on his own initiative and said, ‘I need help now or someone has to help me.’ So I don’t see him as a weak person just because he has depression or because I’ve seen him cry. Quite the opposite.” (08, pos. 100)*

One participant explicitly draws on male stereotypes to make a distinction, referring to the partners’ job and participation in the household:

*“He’s a primary school teacher, so he’s not the typical man who, I don’t know, is a car mechanic and doesn’t do anything around the house. Well, he’s never been that. So in that respect, he’s not the stereotype anyway (laughs).” (11, pos. 112)*

### 2. Positive impacts on relationship

Some participants reported that the illness has provided the opportunity for their relationship to grow in a positive sense, such as causing them to be more sensitive to the partner’s condition and both partners having more empathy for each other. One participant who had herself suffered from depression remarked:

*“Now I would say that we have gotten stronger, we have grown together.” (05, pos. 219)*

In this regard, one participant mentioned the need to ensure that she allowed her partner to take responsibility for looking after himself, describing this as a challenge:

*“More open, even more trusting and considerate of one another. Both of us, in relation to each other. And also more attentive. So again, both of us to a certain extent. But then I also had to step back a little so that I didn’t become too attentive towards my partner, but rather made sure that I looked after myself and attended to my needs and trusted that my partner was attentive enough to be attentive to HIMSELF and to let me know if he needed or didn’t want something. To be able to stop at some point and leave people to their own devices.” (07. pos. 46)*

Many participants particularly emphasized the improvements in their partner’s behavior within the relationship and their communication patterns following therapy. One participant, for example, described how her partner now talks more openly about certain topics following therapy and reflects openly and critically on conversations and his behavior:

*“He’s just become more open now. He might talk about some things differently than before. Or he talk more about certain things in general. Yes, or that he also [...] says things like ‘yes, crap, maybe I shouldn’t have said that’ or ‘I shouldn’t have reacted like that.’ And that sort of insight didn’t really exist before.” (14, pos. 101)*

Another participant described the positive development of her partner’s self-perception and his ability to express his feelings:

*“He thinks more about himself. In a positive sense. Much more selfish in his decisions about what he wants to do. I think that’s good. [...] he always used to let himself be pulled along by others and now it’s more that he also says what he doesn’t want and what he does want. Which I think is really good.” (01, pos. 66)*

### 3. Burdens and challenges in coping with partners’ depression

The participants described various burdens and challenges in dealing with their partner’s illness. These differed depending on their circumstances and the course of treatment.

#### 3.1. Seeking help and the beginning of treatment.

Participants reported a range of support measures they provided their partners ahead of treatment, such as accompanying him to treatment, driving him to the hospital, or having consultations with physicians. When describing their partners’ journey towards treatment, participants often referred to situations that served as tipping points and lead to (inpatient) psychiatric treatment. Such situations included aggressive escalations during couples’ disputes, partners experiencing suicidal thoughts, and partners having breakdowns at work:

*“Then he had a major breakdown. It was actually a small job-related issue where something didn’t work out. He then had another one of his crazy outbursts and then at the end of these outbursts he was in tears. And then I just grabbed him and said it CAN’T go on like this. You need to get some help now.” (2, pos. 14)*

With a few exceptions, almost all participants highlighted the difficulties in finding an available therapist or psychiatric hospital when it was needed:

*“He would have gone to therapy earlier at times, if it hadn’t felt like he needed five signatures for everything─fill out this form, then something’s missing, and then he has to go back again. […] If the process takes forever and becomes frustrating, and you know: well, do I take this one* [the therapist] *or─if I don’t, then I’m looking at another year of waiting and the next waiting list, just to see whether that one might be a better fit.” (09, pos. 24)*

Many participants expressed criticism and a lack of understanding of the mental health treatment system, referring to the stress and uncertainty the prolonged search and wait for an available therapy slot caused for participants and their partners. One participant felt overwhelmed and heavily burdened at the time of her partner’s admission to hospital:

*“I couldn’t take it anymore. I was so exhausted. I no longer knew— because it was always just so—we couldn’t carry on this way and I was always on sick leave, then there’s not enough money and I felt that now I was ruining everything. […] And yes, he didn’t do so many things anymore. I had to initiate everything myself.” (14, pos. 13)*

Accordingly, another participant described the relief she felt after her partner was hospitalized:

*“For me it was more relaxed at this point, because at the start he was away for a whole week. And he wasn’t allowed to come home in the first two weeks. […] Then I’d calmed down a bit and I think he had too, of course.” (04, pos. 78)*

Although most of the participants reported a willingness on the part of their partners to seek treatment, they also described taking an active role in the treatment process themselves. This involved persistent efforts to raise the topic and encourage help-seeking behavior over an extended period. One participant, for example, recounted how she repeatedly addressed the need for treatment before her partner acknowledged the problem:

*“Before he took action, I had long been saying that he needed psychological help. And he always denied it.” (05, pos. 56)*

Another participant emphasized her own initiative in actively facilitating the first step toward professional help:

*“And then I said: ‘Just go to the general practitioner and tell him that.’ […] So it was actually MY initiative, if you like.” (10, pos. 12)*

In contrast, some participants reported that their partners had made the decision to begin treatment on their own. The following quote is from a participant who had previously had positive experiences with therapy herself. Still, she hesitated to suggest treatment to her partner, as it was important to her that the decision be made independently by him:

*“But I refrained from giving any advice. It was actually more like something my husband announced to me, just like: ‘I would like to do that, by the way.’ And I was surprised, positively so, but it also showed me that he was thinking many steps in advance.” (07, pos. 10)*

#### 3.2. Lack of understanding among female partners.

The participants often described difficulties in understanding and sympathizing with their partner’s behavior. According to the participants, the way in which their partners talk about their depression makes it difficult for them to understand the illness. One participant was critical in describing the way her partner explains his feelings by resorting to the same old phrases:

*“With him it sometimes seems like a description of things sourced from the Internet instead of a description of what he has experienced himself.” (09, pos. 70)*

The participant, who reported that she was very stressed due to increased responsibilities around her job, the household, and their children since the onset of her partner’s illness, expressed her “difficulty in understanding” her male partner’s behavior. The following quote illustrates the participant’s perception that her partner does not adequately fulfill his share of family responsibilities, despite prior agreements. She exemplifies this by describing a situation in which he invests energy in his training but lacks the strength to complete agreed-upon household tasks afterward. However, it remains unclear whether her comment reflects a lack of understanding of the illness or dissatisfaction with what she perceives as an unequal allocation of effort:

*“It’s just so incredibly variable, things that you’ve arranged with the gardening and, he has tennis training and then the garden is on the agenda and then he goes to tennis training but can’t do the garden anymore, because then it’s time for bed and being depressed. And that’s just, um, difficult to understand.” (09, pos. 38)*

Another participant who has experienced aggressive behavior from her partner described her attempt to acknowledge the aggressive behavior as part of the symptoms and not to take it personally. She ultimately attributed the difficulty in reconciling her partner’s behavior with her ideas of what a relationship should entail to her inadequate understanding of the illness:

*“How can someone be like that to me, who says he loves me and then doesn’t behave as if he does? So it’s incredibly difficult for me not to take it personally. [...] And yes, but I already know that it’s probably a mistake on my part, that I have to accept it more, that it really is an illness. But it’s just hard when you’re treated badly again and again.” (13, pos. 47 + 71)*

#### 3.3. Concerns due to the clinical picture provided and the consequences of the illness.

Participants frequently described concerns regarding the illness. In this regard, they often referred to the duration of the illness as well as symptoms of depression and their consequences, such as suicidal thoughts:

*“Of course there is also the concern of whether we will catch it in time if he falls into a hole again, of overlooking something. […] So he promised me that he wouldn’t do something to himself, but you never know for sure. And yes, this worry about him is still there.” (02, pos. 26)*

Against the backdrop of these concerns, participants described their need for honest and meaningful communication with the partner. One participant described being concerned as to whether her partner was capable of recognizing his limits in good time and communicating them:

*“I still rely on him letting me know how he is doing and what he needs. Sometimes I worry about whether certain things are in fact overwhelming for him or that he’s lets me know too late that they’re overwhelming for him.” (07, pos. 18)*

Another participant expressed the conflict between her need to be informed about her husband’s condition and conversely his need to not talk about it:

*“Very open now. And I think that’s very good. He even told me I was asking too many questions. That’s why I’m now trying to ask a little less. Which is difficult for me. I find it difficult, especially now in the darker time of year. That’s when things tend to get worse. And so it’s not quite enough [information] for me. But I try to respect his needs and not ask so often.” (01, pos. 44)*

#### 3.4. Monitoring the partner.

Some participants described being concerned that they would not notice symptoms and be able to react in time if they became exacerbated. Out of concern for their partnes, they reported taking on responsibility for them. One participant reported having monitored her partner over the weekend. This occurred just before he was finally admitted to the psychiatric clinic, after initially being sent home despite a suicide attempt:

*“And then I thought to myself, well, if I were to tell someone who is planning to kill themselves that he should promise me that they won’t kill themselves until Monday… I don’t know if that would work. But well, then I more or less took him under my wing and didn’t let him out of my sight, basically.” (04, pos. 76)*

Another woman described ambivalent feeling regarding the assumption of responsibility for her partner. On the one hand, she expressed deep fear of a possible suicide attempt. The effort to prevent this by monitoring her husband puts her under considerable pressure. On the other hand, she reported significantly restricting her own social life in order to be able to provide this care for him:

*“And I also realize that I’m locking myself in the house. Maybe to, well, what does it mean to control him? I’m just afraid that he’ll hurt himself again or do more this time. And then I always think to myself, well, I’d better not go out at night. I don’t want him to hurt himself again. So I put a lot of pressure on myself. Probably more than I need to.” (06, pos. 114)*

#### 3.5. “Shouldering more responsibility”.

Most participants described taking on additional responsibility that led to increased working hours and time spent on household chores.

*“Everything depends on me. Because they* [the children] *just know, you can’t rely on dad. The children know I’m the one who gets up in the morning, who definitely wakes them, who definitely makes breakfast. I definitely take care of everything. […] And I’ve already increased my working hours to somehow, well, raise my share of the income and our savings in case there are further setbacks.” (09, pos. 52,56)*

Some of them considered taking on additional responsibility as self-evident and did not question the changed division of labor. In such cases, participants often referred to the amount of work the man continued to carry out despite the illness:

*“He also got some time off towards the end and then he came home and did something in the garden. […] That was also on his own initiative. But that was also a time when he was feeling better. Yes, there were never really any problems and nothing has really changed. So neither where it was so bad or where it really worked.” (14, pos. 61)*

Other participants justified the extra work by saying that their partner acted in the same way in the past when they themselves were ill and their partner had to take over all responsibilities:

*“I honestly didn’t think about it. I just did it. So there was no discussion at all. I mean, it was similar, uh, when I had my cancer. If I couldn’t do something, then he did it. So with us it’s simply a situation of where if you’re unwell, you’re unwell. Period. And so I didn’t even think about it.” (05, pos. 156)*

In contrast, other participants reported being overloaded as a result of taking on additional responsibility. This was reported more frequently by participants with younger children and by participants who reported having the impression that their partner is not sufficiently involved or does not value their efforts:

*“I always keep the family going, so to speak, but [I am] also totally involved in my job. Of course, I have to deal with the children a lot. Then there is a lot to do in terms of housework, so that’s very important. And when I’m no longer able to do it, then it’s just, well, then it’s even worse. So if I show weakness, so to speak, then he’s completely useless. Then, yes, he simply puts his feet up, and nothing works anymore, and when I get so frustrated and just can’t cope with it, to the point where I say I just need a little help, then, yes, there are many, many, many dumb situations. There’s a lot of aggressive behavior. So when I accused him of something or my frustration came across to him as an accusation, then there was aggressive defensive behavior. Then he lobbed accusations at me.” (09, pos. 8)*

The participant described the need to compensate for the load in some way in order to get through the situation:

*“Of course, it’s more exhausting when you simply shoulder more of the burden. It’s a really shitty situation because you’re just so affected. But you can’t do anything about it. That’s, yes, so of course it drags you down too. So you have to look at how you can somehow keep yourself on track. So it’s very, very, very, very exhausting.” (09, pos. 76)*

One participant, who has been supporting herself on her own since her partner fell ill, expressed her longing for peace and the opportunity to spend some time alone:

*“So I realize that I sometimes push myself to my limits. I think, my God, you come home from work, and it’s really exhausting. And then I come home and I think, I just want to have some REST, right? Like this. I’m glad when he’s not home. When he goes somewhere or something, so that I just have a few hours to myself.” (10, pos. 54)*

#### 3.6. Reflecting on and adjusting their own behavior.

Participants reported reflecting on their own day-to-day behavior with regard to its possible effects on their partners’ mental health:

*“So I’m also more concerned about: how is he? What do I say? What do my statements lead to? What do they trigger in him? What can I do to make him feel better? What have I done to make him feel worse? Thoughts like that. So it was more carefree before the depression.” (01, pos. 42)*

Participants who reported having conflict-heavy or violent relationship dynamics particularly described efforts to avoid conflicts in their relationship by holding back criticism or refraining from asking their partners to help with household tasks. One participant explained how carefully she monitored her own speech in order to avoid conflict:

*“You have to walk on eggshells aroung him. I have to be very careful with what I say. If I speak my mind, then I’m in trouble straight away.” (06, pos. 60)*

Another participant described how such patterns led to a broader reconfiguration of relational expectations. Through repeated compromises and reduced expectations, she and her family sought to minimize the likelihood of conflict escalation:

*“We made quite a few compromises, so to speak. Or kind of as a concession, like: okay, then it just won’t work. So expectations are much, much, much, much lower. I think. Yes, because in the past, there used to be conflicts, when I had expectations or the children or someone else expected something and it didn’t happen, then yes, you just say so. And then it became a conflict. And then, when it escalated, you thought it was because of some pointless shit or some little thing. So we kept dialing it back, so to speak, trying to reduce the potential for explosion to a very low level.” (09, pos. 54)*

Some participants reported restricting their free time activities and social contact with others due to their partner’s illness. They described their partners as being less sociable and interested in meeting other people or as being unable to cope with the great deal of socialization required at get-togethers and celebrations. In the following quote, one participant reports feeling guilty about going out in the evening:

*“He didn’t deal with it well when I was away, even in the evenings with friends or something. Which was stressful for me, because you actually want to have fun, but then you feel guilty because you know that your partner is having a bad time.” (03, pos. 42)*

In the following quote, a participant describes the loss of things that give her pleasure in life as a result of adjusting to her partner’s needs. She concludes her story with the desire to distance herself from her partner in order to gain a self-determined living:

*“I have simply adapted myself, permanently, and actually subordinate my needs. So all the things that make me happy in life are as good as gone. I only conform to him [...] But I can’t always give in. I, I, I always make myself smaller. And I don’t just live to fit with his life. I also have my own life.” (13, pos. 8)*

#### 3.7. Thoughts of separation.

Some participants brought up the subject of separation during the interview. These women often reported physical or verbal violence in their relationships. They discussed the possible option of a separation, weighing up self-protection and protecting their children against the love they feel for their partner and the hope that things would go back to the way they used to be. One woman reported being worried that a separation would lead to a worsening of her partner’s depression symptoms and increase the risk of suicide, making her hesitant to pursue a separation:

*“But of course I’m also afraid that if I break up with him, it will be even more of a reason for him to fall even deeper into depression and maybe do something to himself. And I don’t want to be blamed for that.” (13, pos. 49)*

Some of these women mentioned expectations from third parties regarding the possibility of separation. They received both the recommendation to separate from and the expectation to stay with their partners, both of which they described as inappropriate. In the following example, one participant states that her partner was afraid of being abandoned, while her mother-in-law appreciated the fact that she stayed with her partner despite his depression, both of which point to the potential disconnect between suffering from mental illness and the ability to maintain a relationship. However, the woman rejects the possibility of separation as a consequence of the illness:

*“These are things that I take go without saying* [staying with her partner)]. *I talked to his mom a lot during that time, for example. And she also said that it was nice that I was still there for him, that not everyone could do that, that it isn’t easy. And it often wasn’t easy either. But just because it wasn’t easy, I never thought about it being expected of me or something, if I had wanted to—this might sound stupid, but I could have left at any time. So that was also a worry he had. When he had the breakdown, for example, he just told me not to leave him on his own, that I should wait for him and that he was sorry. That he was the way he was. And. That he was afraid he’d lose me because he was ill. Which was never up for debate for me. But it’s still something that bothers him.” (08, pos. 68)*

### 4. Experiences with and expectations of support

#### 4.1. Joint therapy session during treatment.

Participants were asked about their attitudes and experiences concerning joint therapy session during treatment of their partners. During the treatment, one joint therapy session with the partner was regularly provided. As reported by the participants, such sessions were sometimes offered by the clinic or the therapist, but sometimes only took place after they had actively requested them. Some participants reported that they found it a shame that so few conversations took place. Most of them found the opportunity to talk during the therapeutic process very important and described themselves as being irritated when such an opportunity did not exist. The participants found couples therapy useful for bringing their own perspective into their partner’s therapy and evaluated the description of their partner’s situation without their own perspective as being inadequate:

*“I mean, who goes somewhere and says ‘okay, I shout at my children and my wife at home,’ no-one normally does that. You don’t feel great yourself anyway. Then you don’t go somewhere and present it like that, but then you get those depression symptoms that everyone knows, like, yes, I’m listless, I don’t really know what to do with myself, I’m not very sure of myself, and somehow I don’t really feel anything and so on [...] as if half the clinical picture was actually missing. In terms of the symptoms, and nobody was interested in that. So they were formulating a diagnosis and the whole time they wanted reports that were 25 years old and older. But they didn’t want to talk to someone who was with him all day. That was, yes, strange.” (09, pos. 28 + 34)*

In addition, participants appreciated that the couples therapy sessions created a setting in which intensive discussions between both partners could take place, allowing them to address couple dynamics or the sources of misunderstandings. In this regard, they valued the presence of a neutral third person during the discussion:

*“Simply that you had a space where there was someone who was also neutral, yes, and could perhaps explain the partner better. So either his point of view or my point of view. That way it didn’t escalate, but you could think about what the [therapist] had said about it and then reflect on it better. That’s how I felt about it. Yes, I found it very pleasant. And you were perhaps more likely to dare to say something without worrying about it, yes, getting out of hand or being taken the wrong way, because you had this neutral person who perhaps filtered everything a bit.” (05, pos. 275)*

Other participants described how they were grateful for the opportunity to express their own perspective and their experiences of living with their partner. The following example shows a woman who describes herself as being highly stressed due to her domestic situation and who had been diagnosed with burnout during her partner’s period of depression. In this case, the therapist called from the hospital, with the husband present, for a couples therapy session. The participant valued the opportunity to talk about her own condition and ultimately went to a therapist herself to meet her need for supportive conversations:

*“I thought it was good. That was the first and only time that I had my say and that someone asked me how I actually felt about it. Because my husband was never interested in that either. It was always just about him. He’s in a bad way, he’s in a bad way and he still doesn’t see how much his wife is suffering. Or he doesn’t want to see it. That’s why* [to have more conversations] *I have my own therapist now.” (06, pos. 74-78)*

However, a few participants felt that they do not see any reason for couples therapy because they regard the illness as the partner´s matter. In this way, these participants distanced themselves from the illness and the therapeutic process. In the following example, one participant justified her unwillingness in taking part in the review by referencing own difficult experiences, which she managed to get through herself:

*“Yes, probably, at least that’s how I think about it now: I didn’t have it easy or have it easy now either because there were situations that were difficult for me in the year before and I just, yes, pulled myself together and said that everything would go on. Oh and somehow, I just didn’t want any of it. I didn’t want to speak about myself so much either. Because I thought, it’s my husband who should be treated and please just leave me alone. [...] They also said: ‘it might help you too’. But I just said: ‘nope.’ I just didn’t want it.” (14, pos. 41)*

#### 4.2. (Professional) support for female partners.

The participants were asked to what extent they had used support services from within both their social environment and professional context and to what extent these were helpful to them. The most common reason for using professional and private support was to have someone to talk to. In a private context, participants valued the support of friends and family members for both relief and advice:

*“So I have a circle of friends and I talk to them about it. Of course I do. Since I need that.” (10, pos. 66).*

Some participants made use of psychotherapy themselves in order to have professional support during their partner’s illness or to process the events in retrospect:

*“When I started working, I realized again that there was something wrong with me that I couldn’t sort out and that I was simply undergoing treatment for myself and working through what had actually happened and what I was carrying with me, which I didn’t necessarily see as an extreme burden at the time. Probably as a kind of protective gesture, because I knew the family had to keep going. But afterwards, I came to terms with it to a certain extent, yes.” (07, pos. 66)*

Some participants stated that they had not sought professional support, which they justified by saying that their own need for help was not so large or not sufficiently great to make use of professional support:

*“Well, if it gets worse, I can imagine that* [making use of assistance]. *Yes.” (10, pos. 86).*

Other participants with a need for support described having a difficult time finding support options tailored to their situation or needs, or described lacking the time to initiate any kind of professional support or to apply for the help they think they actually need:

*“And that […] would have meant even more work for me at the time. I had to see where I could go and how I could fit it into my schedule. And yes, perhaps I shied away from the effort and also thought, ‘no, like that’s really going to help me?’” (14, pos. 71)*

## Discussion

This study examined the challenges and consequences of living with a depressed man from the perspective of their female partners, with a particular focus on the couple’s relationship, the way in which the partners deal with the illness, and their need for support. Female partners were found to be highly involved in the treatment process, in which they take on an active and demanding role, particularly at the outset of treatment. They also view their experiences with their partner as an important source of information for mental health professionals regarding their partner’s symptoms and behavioral patterns. Therefore, they consider it beneficial to be involved in the treatment process. However, the experiences of participants showed that female partners are only involved in the treatment to a limited extent. The participants also found it incomprehensible that their experiences with their partner were given only little consideration. These results confirm results of Priestley et al. (2016), which also found that relatives felt that they were not sufficiently involved in the treatment process and also received little support for their own situation [8]. Furthermore, the participants of this study criticized the difficult access to psychiatric care, seeing it as a major burden that delays the treatment of their male partners.

Participants described a variety of caring behaviors and responsibilities they undertook with respect to their partners during the course of their treatment. These include considering the effects of their own behavior on their partners’ mental state or keeping stress away from their partners in everyday life. However, shouldering more of the load may lead to an overload of men’s partners, particularly if there are children in the family or if the partner must bear sole responsibility for the couple’s financial security. In this study, participants who described their experience of taking on additional work and responsibility as stressful also reported a severe or long-lasting illness of their partners and that they find their partners’ efforts to cope with the illness inadequate or do not feel that their partners appreciate their efforts. Our findings corroborate other studies regarding significant demands placed on female partners, including a responsibility to “save” the partner or to avoid conflicts [2,6], as well as feeling the need to restrict their own social life so as not to leave the partner on their own, especially in cases where they felt there was a risk of suicide [6]. Female partners of men with depression take on additional tasks, often over an extended period of time, an experience that is largely perceived as stressful. At the same time, they are unsure about how much cooperation they can or should demand from their unwell partner. This aspect is also evident in another study where female participants of men with depression critically reflected on the division of labor in their relationship along stereotypical gender lines [21].

Generally, the results of this study indicate that participants are committed to understanding the behavior of their partners. In this regard, they value good communication as a prerequisite for being able to respond to the needs of their partners as well as an essential basis for successful treatment. In light of this, they demand that partners be able to communicate their feelings to them and their therapists and criticize men for poor communication patterns that they consider to be insufficient. However, maintaining good communication between couples during the illness phase is challenging for both partners [2]. Some couples were found to successfully cope with the illness and even emerge with stronger relationships under certain conditions, including a high quality of relationship to begin with, the availability of treatment, a shorter duration of depressive episodes, less severe forms of depression, and adequate support [7].

This aligns with our findings that the type of support most readily accepted by the participants from the professional system is joint therapy sessions undertaken as part of the male partner’s treatment. Aside from this, the women in our sample generally tended to make little use of professional help, as it often did not meet the women’s actual needs or the barrier presented by the additional organizational effort required was considered too high. Instead, the great need for conversations, as expressed by many participants, is already met by close contacts from their personal network.

Traditional norms of masculinity served as a point of reference in the participants’ descriptions of the men’s behavior within the relationship and how they dealt with the illness. In this context, participants positively evaluated characteristics in men that do not correspond to stereotypical norms of masculinity, such as higher emotional capacity, both in relation to coping with the illness and in shaping the relationship. Instead, they characterize their partners as possessing attributes of alternative masculinities, such as by pointing out that their partners are “non-typical” men. Women who described their partner’s behavior and symptoms of depression with characteristics that correspond to stereotypical norms of masculinity also reported that they had a very tense relationship and described themselves as being very stressed and insecure, especially in relation to their partner’s aggressive behavior. At the same time, these symptoms are categorized by participants as part of the clinical picture of depression. In this regard, participants alternate between showing understanding for the partner’s behavior and seeking to preserve their sense of self-worth, which cannot be reconciled with the acceptance their partner’s behavior. By referring to the significance of norms of masculinity in the way that men deal with the illness, the participants are in line with the current discourse on the significance of stereotypical male behavior in depressive disorders [11,25]. This is consistent with findings from prior research, which indicate that traditional masculinity norms influence not only men’s help-seeking behavior [11], but also how mental health professionals interpret male patients’ symptoms and engagement with treatment. Traits that deviate from traditional masculinity norms are seen as more helpful in managing the illness [16]. However, recent research indicates that men’s coping strategies are shaped by a broader spectrum of gender norms [18]: Some men with depression actively reinterpret masculine norms, using them to promote self-determined and proactive coping strategies [19].

### Strengths and limitations

By examining the challenges and consequences of depression in men from the perspective of their female partners, this study provides important insights into the relevance of a gender perspective in mental health research. It contributes to a better understanding of how gendered relationship dynamics and masculinity norms influence the experience and management of male depression within intimate partnerships. However, our recruitment stretegy of participants may have influenced the sample composition. Sufficient time capacities and technical requirements were needed for participation. Our study sample contains white female participants with sufficient knowledge of German (Migration background or length of stay in Germany were not recorded) and differed with regard to socio- economic aspects. Therefore, our results are based on the experiences and views of the study participants. Female partners of men with depression who have different biographical backgrounds may express different views based on their own experiences. The results of the study also point to the importance of gender norms in dealing with the illness. Therefore, expanding the research question to incorporate the perspectives of same-sex couples could result in greater knowledge of the significance of gender norms regarding coping mechanisms and mental illness.

Further, we conducted Interviews exclusively online via zoom. Interview procedure and feedback from participants gave reason to believe that this kind of setting did not influence the conduct and progress of the interviews. However, because no interviews were conducted in a face-to-face setting, there is no way to compare the interview situations and a possible influence of the setting cannot be ruled out with certainty.

## Conclusions and practical implications

Based on the results of the study, implications arise regarding the mental health treatment of men with depression and the need for further research: joint therapy sessions, as part of the male partner’s overall mental health treatment, seems to be an important measure with regard to psychoeducation. The challenge of making other people understand what depression feels like could be addressed within the professional support system by helping both partners to communicate with each other and better understand the clinical picture of depression. In addition, joint therapy sessions offer a neutral space to discuss problematic aspects of the relationship and to support couples in overcoming the situation. Therefore, it is worth examining whether joint therapy session is given sufficient attention during the treatment period and if sessions address the specific needs of both partners in the context of a male partner’s illness and the female partner’s role. The insufficient assessment and consideration of the home situation through mental health professionals as perceived by participants could be addressed by considering the family situation and potential needs of support for men and their partners more than before, particularly with respect to male patients with symptoms that are associated with male depression. Additionally, there is a need for lower-threshold services that better align with the needs of female partners, as these needs may not be sufficiently addressed. Furthermore, health services research exploring involvement of female partners in the treatment process could investigate the possibilities and potential benefits of such treatment models in depression therapy.

## Supporting information

S1 Table Interview guide. (DOCX)
