# Supplementary material for: “You have to walk on eggshells around him”: Female partners’ perspectives on the opportunities and challenges of dealing with a male partner’s depression—A qualitative study
Source: PLoS One. 2025 Nov 7;20(11):e0336209. doi: 10.1371/journal.pone.0336209 (PMC12594359; doi:10.1371/journal.pone.0336209)
Supplement: S1 Table — (DOCX) [file pone.0336209.s001.docx]

| **S1 Table. Interview guide** | | |
| --- | --- | --- |
| Impulse question | Possible requests | Topic / Background |
| **Start of treatment & subjective theory of illness** | | |
| **Would you start by telling me how it all came down to your partner's depression?** | How did you find out that he was depressed?  How or in which way did your partner tell you about the depression? | Narrative conversation entry  First perception of illness  Orientation on masculinity |
| **How did it come about that your partner started treatment?** | Did you motivate or support your partner to start treatment?  If yes: In which way did you support your partner?  Have you been worried about anything regarding treatment? | Enabling / inhibiting factors  - Motivation  - challenges  - Social environment  Orientation towards masculinity; attitude towards treatment |
| **What did you think or what went through your mind when you first heard that your partner had been given a diagnosis on depression?** | How do you feel today about the fact that your partner has a diagnosis on depression?  *If the other person does not identify with the diagnosis:*  You just said that you do not agree with the diagnosis/.... What would be more accurate? What would describe it better? | Attitudes towards the diagnosis and form of treatment  - image of masculinity  - Social expectations/attitudes  - Thematization / how it is discussed   - - over a period of time |
| **What ideas did you have about psychotherapy or psychiatry before treatment?** | Have you had any contact with psychotherapy or psychiatry? If so, what experiences did you have? |  |
| **What do you think about the cause of the illness / situation / depression?**  *Orientation on the wording of the other person* | Do you see any other causes?  What do you understand by healing in the context of depression?  - How do you recognize it?  - How would you measure this? | Subjective theory of illness / explanatory models  (e.g. biological/sociological) |

| Impulse question | Possible requests | Topic / Background |
| --- | --- | --- |
| **Dealing with the disease in a partnership and close environment** | | |
| **How would you describe the relationship between you and your partner since the start of the illness?** | What was it like before? Did anything change in your relationship as a consequence of the illness, for example emotionally, socially or sexually?  How do you both talk about the illness? | Division of roles / changes due to the illness |
| **When you think about the roles and tasks in your relationship - to what extent have these changed as a result of your partner's illness?** | For example in the area of  - the care of children,  - earning money,  - with regard to social contacts  To what extent has the diagnosis or treatment changed how you experience your partner as a man?  If your partner is a man, do you see any differences to heterosexual couples in this regard? | *Division of roles / changes due to the disease*  *Gender regimes* |
| *If family:*  **To what extent does the illness affect everyday family life?** | How would you describe the way your family deals with the disease?  Did the treatment change anything in this respect?  Would you say that the depression affects or has affected the children? In which way?  How do you think your children are coping with the illness? What is going well, what is going less well? | Disclosure to children  Parentification  Offers of help for children |

| Impulse question | Possible requests | Topic / Background |
| --- | --- | --- |
| **Course of the disease / treatment / therapy** | | |
| *Depending on the interviewee's framing, talk about treatment and/or therapy*  **Now we would like to talk about your previous impression regarding your partner's treatment.**  **What were your expectations before**  **the treatment / therapy?** |  | Expectations before therapy |
| **To what extent did the treatment/therapy lead to changes?** | In relation to:  - their relationship to each other  - the relationship to the children   - - other aspects that are important to you |  |
| **Which role do medications play**  **in the treatment?** | Have you discussed taking medication with your partner?  What is your opinion on medication? | Medication |
| **Did conversations with relatives take place during treatment?** | *If yes:* How do you feel about them?  *If no:* Why not?  Would you like to have more conversations? |  |
| **How do you feel about it when your relationship is discussed in therapy?** |  |  |
| **What do you wish for with regard to**  **treatment or the further course of therapy?** | Are there certain topics that are particularly important to you?  What would you have liked differently? | Treatment requirements |

| Impulse question | Possible requests | Topic / Background |
| --- | --- | --- |
| **Social environment & disclosure** | | |
| **How does your environment deal with your partner's illness / situation / depression?**  *Orientation towards the wording of the person you are talking to* | What role does the disease play in your family/acquaintances/friends?  Which expectations were put on you as a partner?  Do you experience support in dealing with the illness and if so, what kind? (e.g. from partners, friends, etc.)  Do you have contact with other partners in a similar situation? | Dealing with the environment during therapy (family, friends, peers, acquaintances):  - Reactions (of the environment)  - Support  - Changes regarding relationships and possibly partnership   - - Stressful aspects |
| **Do you talk to anyone else apart from your partner about the illness/depression/therapy?** | If so, with whom?  What experience have you had so far?  If not, why not? Who do you not talk to about it?  How do you react when you are asked to talk about your partner's illness?  Have there been situations in which you had to tell other people about the illness instead of your partner? | Disclosure/confidentiality  - Context (job, family, friends)  Reasons for disclosure/concealment  - Health behavior  - Stigma  Positioning / self-positioning in relation to masculinity orientation |

| Impulse question | Possible requests | Topic / Background |
| --- | --- | --- |
| **Self-care / support** | | |
| **How did your life change as a result to your partner's depression?** | To what extent were these positive changes?  To what extent were these negative changes? |  |
| **Have you taken advantage of any support services?** | For example:  - Relatives' groups  - Household assistance  - Online offers  - Coaching  - Support from the social environment  - Therapy for yourself  What kind of support would you have wished for in your situation or what would be (additionally) helpful for you at the moment? |  |

| Impulse question | Possible requests | Topic / Background |
| --- | --- | --- |
| **Gender & Society** | | |
| **How do you think depression in men is perceived in society in general?** | Do you think it is different for women (when they have depression)?  What do you think is the reason for this? | Gender stereotypes, stigma  Representation in the media  Celebrity “role models”  Gender perceptions in the context of illness |
| **Has the diagnosis or treatment affected you in any way in the way you think about people with depression?** |  | Stigma |

| **Conclusion** | | |
| --- | --- | --- |
| **What are your wishes for the future?** | What do you wish for yourself?  What do you wish for your relationship / for yourself and your partner? | Prospects |
| **Is there anything else that is**  **important to you that hasn't been mentioned yet?** |  |  |
